# Dynamics of Glass Forming Liquids with Randomly Pinned Particles

**DOI:** 10.1038/srep12577

**Published:** 2015-07-24

**Authors:** Saurish Chakrabarty, Smarajit Karmakar, Chandan Dasgupta

**Affiliations:** 1 Centre for Condensed Matter Theory, Department of Physics, Indian Institute of Science, Bangalore, 560012, India; 2 TIFR Center for Interdisciplinary Science, Narsingi, Hyderabad 500075, India; 3 Jawaharlal Nehru Centre for Advanced Scientific Research, Bangalore 560064, India

## Abstract

It is frequently assumed that in the limit of vanishing cooling rate, the glass transition phenomenon becomes a thermodynamic transition at a temperature *T*_*K*_. However, with any finite cooling rate, the system falls out of equilibrium at temperatures near *T*_*g*_(>*T*_*K*_), implying that the very existence of the putative thermodynamic phase transition at *T*_*K*_ can be questioned. Recent studies of systems with randomly pinned particles have hinted that the thermodynamic glass transition may be observed for liquids with randomly pinned particles. This expectation is based on the results of approximate calculations that suggest that the thermodynamic glass transition temperature increases with increasing concentration of pinned particles and it may be possible to equilibrate the system at temperatures near the increased transition temperature. We test the validity of this prediction through extensive molecular dynamics simulations of two model glass-forming liquids in the presence of random pinning. We find that extrapolated thermodynamic transition temperature *T*_*K*_ does not show any sign of increasing with increasing pinning concentration. The main effect of pinning is found to be a rapid decrease in the kinetic fragility of the system with increasing pin concentration. Implications of these observations for current theories of the glass transition are discussed.

The glass transition is characterized by a rapid increase of the viscosity (*η*) and the structural relaxation time (*τ*_*α*_) with decreasing temperature^1^,^2^,^3^,^4^. Recent progress^5^,^6^,^7^,^8^,^9^,^10^,^11^,^12^,^13^,^14^,^15^,^16^ in understanding various dynamical aspects of this phenomenon has shed some light on this subject, but the question of whether an “ideal” thermodynamic glass transition, signaled by the vanishing of the configurational entropy density, can occur at a temperature lower than the experimentally defined (dynamic) glass transition temperature remains unanswered. Recently, it was proposed in Ref. 17, that the difficulty in observing the putative ideal glass transition in simulations and experiments can be bypassed by considering liquids in the presence of quenched disorder and studying the effects of varying disorder strength on the thermodynamic and dynamic properties of the liquid. It was argued from Mean Field (MF) and Renormalization Group (RG) calculations, that a thermodynamic glass transition at a temperature higher than the transition temperature of the liquid without disorder can be achieved by increasing the strength of the quenched disorder in the system. These studies assumed that the Random First Order Transition (RFOT) theory^3^,^4^,^5^, which is currently the most popular theoretical framework for describing equilibrium and dynamic properties of glass-forming liquid near the glass transition, remains valid in the presence of quenched disorder. However, the validity of the RFOT description is still controversial. An alternative description^18^ of glassy dynamics, based on the behavior of kinetically constrained systems, does not show^19^ the occurrence of a transition at a non-zero temperature with increasing disorder strength. Therefore, a numerical investigation of whether this transition actually occurs in model glass-forming liquids would help in determining which of these competing theories is better at describing structural glasses. The possibility of observing a thermodynamic glass transition in simulations and experiments relies on the theoretically predicted increase of the transition temperature with increasing strength of pinning. It would be interesting to check by simulations whether this prediction is valid for three-dimensional glass-forming liquids. A numerical study of glassy behavior in pinned liquids would also help in understanding the results of a recent experiment^20^ on colloidal systems with the kind of pinning disorder considered in the theoretical studies.

Existing simulation results for the effects of quenched disorder on the dynamics of supercooled liquids^13^,^21^,^22^,^23^,^24^,^25^ were obtained using different ways of generating the disorder. In this study, we consider the disorder generated by randomly choosing a fraction *ρ*_*pin*_ of the particles from an equilibrium configuration of the supercooled liquid at temperature *T* and freezing them in space. We henceforth refer to this geometry as the “random pinning” geometry. In these systems, the potential that describes the interaction of a pinned particle with a mobile one is the same as that for two mobile particles. This kind of quenched disorder can be realized in liquids confined in a statistically homogeneous porous medium^26^ obtained by freezing a fraction of the particles in an equilibrium configuration of the same liquid. It can also be realized in experiments on colloidal systems^20^, using optical traps to pin the particles. This way of generating the disorder, considered in Ref. 17, has certain advantages. For instance, preparing an equilibrated system is straightforward, as the state obtained by instantaneously freezing a randomly selected fraction of the particles in an equilibrated liquid configuration is a valid equilibrium configuration of the system with the pinning disorder. In earlier studies^13^,^22^,^24^,^25^, the relaxation time of the system with random pinning was found to increase very rapidly with increasing concentration of the pinned particles. The RFOT description which forms the basis of the theoretical calculations of Ref. 17 predicts a divergence of the structural relaxation time at the putative entropy-vanishing thermodynamic transition. Therefore, the phase diagram in the (*ρ*_*pin*_ − *T*) plane can be obtained by locating the temperatures at which the relaxation time diverges for different values of the pin density. Since a rapid growth of the relaxation time is a defining feature of glassy behavior, the dynamics of pinned liquids is interesting by itself. For these reasons, we have carried out a detailed study of the dynamics for two model glass forming liquids in the presence of random pinning using extensive numerical simulations.

## Theoretical predictions

Before going into the details of our results, we briefly discuss the arguments presented in Ref. 17 about the phase diagram of the randomly pinned system in the (*ρ*_*pin*_ − *T*) plane. In the RFOT theory, a thermodynamic glass transition is characterized by the vanishing of the configurational entropy density *s*_*c*_ associated with the multiplicity of amorphous local minima of the free energy. In Ref. 17, the arguments of RFOT were extended to glass forming system with randomly pinned particles. It is physically reasonable to assume that the configurational entropy of a liquid decreases with increasing pinning fraction *ρ*_*pin*_. In Ref. 17, it was assumed that the configurational entropy density decreases linearly with *ρ*_*pin*_,


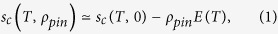


for small values of *ρ*_*pin*_, with *E*(*T*) > 0. In RFOT, *s*_*c*_(*T*,0) is supposed to go to zero at the Kauzmann temperature *T*_*K*_(0), the argument of *T*_*K*_ being the value of *ρ*_*pin*_. Eq. (1) then predicts that for *T* > *T*_*K*_(0), the configurational entropy should vanish at a critical pinning fraction 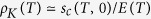 and a thermodynamic glass transition should occur at that pinning fraction. Assuming that this critical fraction *ρ*_*K*_(*T*) < 1, the thermodynamic glass transition temperature *T*_*K*_(*ρ*_*pin*_), obtained from the condition *s*_*c*_(*T*_*K*_,*ρ*_*pin*_) = 0, should increase from *T*_*K*_(0) as *ρ*_*pin*_ is increased from zero. In Ref. 17, a MF analysis was carried out for the spherical *p*-spin model to calculate *T*_*K*_(*ρ*_*pin*_), as well as the critical temperature (*T*_*C*_) of Mode Coupling Theory (MCT)^27^ which represents the temperature below which the dynamics of the system is dominated by activated relaxation processes. It was shown that the dependence of these two temperatures, *T*_*K*_ and *T*_*C*_, on *ρ*_*pin*_ are such that they meet at a “critical” value of *ρ*_*pin*_, at which the thermodynamic glass transition disappears. A real-space RG calculation predicted that the line of thermodynamic glass transitions in the (*ρ*_*pin*_ − *T*) plane has a positive slope and it ends at a critical value of *ρ*_*pin*_. The dynamic transition at *T*_*C*_ is not found in the RG calculation.

## Systems and Methods

The first model glass former we study is the well-known Kob-Andersen^28^ 80:20 binary Lenard-Jones mixture. Here it will be referred to as the **3dKA model**. The interaction potential in this model is given by


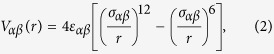


where *α*,*β* ∈ {*A*, *B*} and *ε*_*AA*_ = 1.0, *ε*_*AB*_ = 1.5, *ε*_*BB*_ = 0.5, *σ*_*AA*_ = 1.0, *σ*_*AB*_ = 0.80, *σ*_*BB*_ = 0.88. The interaction potential is cut off at 2.50*σ*_*αβ*_ and we use a quadratic polynomial to make the potential and its first two derivatives smooth at the cutoff distance. Length, energy and time scales are measured in units of *σ*_*AA*_, *ε*_*AA*_ and 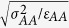, respectively. The temperature range studied for this model is [0.45, 3.00] at number density *ρ* = 1.20.

The second model studied is a 50:50 binary mixture with pairwise interactions between particles that fall off with distance as an inverse power-law with exponent 10 (the **3dR10 model**).


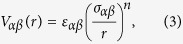


with *n* = 10. The potential is cut off at 1.38*σ*_*αβ*_. We again use a quadratic polynomial to make the potential and its first two derivative smooth at the cutoff. The parameters of the potential are: *ε*_*αβ*_ = 1.0, *σ*_*AA*_ = 1.0, *σ*_*AB*_ = 1.18 and *σ*_*BB*_ = 1.40. The temperature range covered for this model is [0.52, 3.00] at number density *ρ* = 0.81.

We performed NVT molecular dynamics simulations using modified leap-frog algorithm with the Berendsen thermostat. The integration time step used is *dt* = 0.005 in this temperature range. Equilibration runs are performed for ~10^8^ − 10^9^ MD steps depending on the temperature and production runs are long enough to ensure that the overlap correlation function *Q*(*t*) goes to zero within the simulation time. For both the model systems, we performed simulations for *ρ*_*pin*_ in the range [0.000, 0.200] for each temperature. For very low temperatures, we were not able to equilibrate the system for high pin concentrations because of a dramatic increase in the relaxation time in these cases. (See SI for further details).

Dynamic properties are characterized by calculating the self part of a modified two-point density correlation function which we call the overlap correlation function *Q*(*t*) defined as


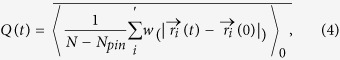


where the weight function *w*(*x*) = 1.0 if *x* < 0.30 and 0 otherwise, 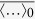 denotes averaging over the time origin and also averaging over different realizations of the disorder, and *N*_*pin*_ = *ρ*_*pin*_*N* is the number of pinned particles. The prime over the summation sign means that the sum is over only the unpinned particles. The use of the window function *w*(*x*) amounts to averaging over the short-time vibrational motion of a particle in the “cage” formed by its neighbors. The value of the cutoff (0.30) used in the definition of *w*(*x*) represents the typical cage size, determined as the square root of the value at the short-time plateau of the mean-square displacement of the particles as a function of time. We average the data over 32 independent runs for each state point and consider systems with *N* = 1000. The *α*-relaxation time *τ*_*α*_ is calculated by the condition *Q*(*τ*_*α*_) = 1/*e*. It is to be noted that *τ*_*α*_-values obtained from the self part of the intermediate scattering function *F*_*s*_(*k*,*t*), calculated at the wave-vector *k* at which the static structure factor *S*(*k*) peaks, are very close to those obtained using *Q*(*t*).

We estimate the MCT crossover temperature *T*_*C*_ by fitting *τ*_*α*_-values for different temperatures for a given value of *ρ*_*pin*_ to a power-law form, 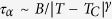 and the temperature *T*_*VFT*_ by fitting the data to a Vogel-Fulcher-Tammann law defined by 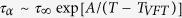. It should be noted that earlier experimental and simulation studies have reported that *T*_*VFT*_ ≈ *T*_*K*_ for unpinned liquids. In the case of the power-law fit, for each value of *ρ*_*pin*_, four to five data points with the highest values of *τ*_*α*_ were used for the fitting procedure. The kinetic fragility is obtained as *K*_*VFT*_ = *T*_*VFT*_/*A*.

## Results

**Figure 1:**
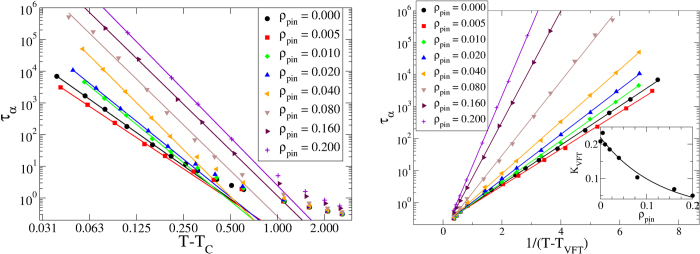
MCT and VFT Fits for 3dKA Model. *Left Panel:* Power-law fits to obtain *T*_*C*_ as a function of *ρ*_*pin*_ for the 3dKA model. *Right Panel:* VFT fits to obtain *T*_*VFT*_ as a function of *ρ*_*pin*_ for the 3dKA model. *Inset:* Kinetic fragility *K*_*VFT*_ as a function of *ρ*_*pin*_. The dramatic decrease in *T*_*VFT*_ with increasing *ρ*_*pin*_ can be clearly seen. The line is an exponential fit to the data.

**Figure 2:**
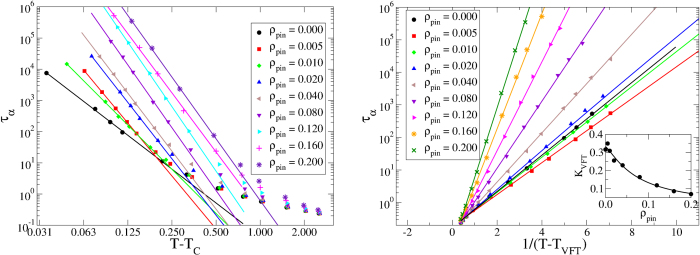
MCT and VFT Fits for 3dR10 Model. *Left Panel:* Power-law fits to obtain *T*_*C*_ as a function of *ρ*_*pin*_ for the 3dR10 model with *γ* = 4. *Right Panel:* VFT fits to obtain *T*_*VFT*_ as a function of *ρ*_*pin*_ for the 3dR10 model. *Inset:* Kinetic fragility *K*_*VFT*_ as a function of *ρ*_*pin*_. The line corresponds to an exponential fit to the data.

Figures 1 and 2 show the MCT and VFT fits for the 3dKA and 3dR10 models respectively. One can clearly see that the VFT fits to the data for both the model systems are excellent, but the MCT fits are not very good over the whole temperature range. If a few data points at relatively high temperatures are excluded in the power-law fitting, a reasonable fit for low-temperature data points is obtained. This gives us confidence about the reliability of the extracted values of *T*_*VFT*_ and *T*_*C*_, although both estimations rely on extrapolation. This, however, is an unavoidable problem in studies of glassy dynamics, affecting both numerical and experimental investigations.

**Figure 3:**
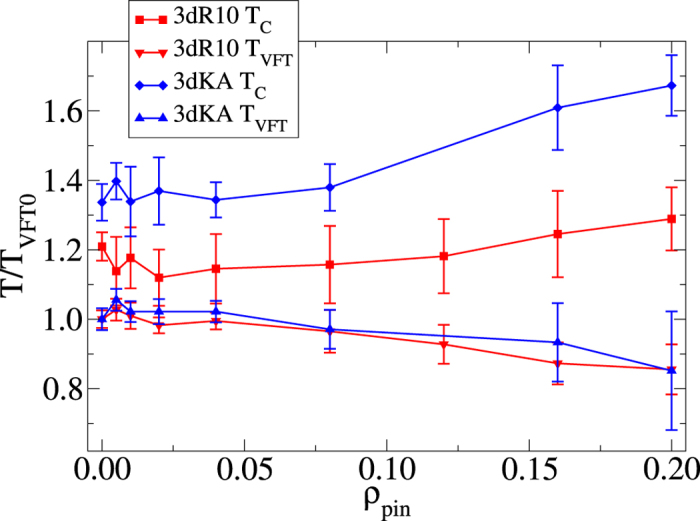
Phase diagram —Variation of the MCT-transition temperature *T*_*C*_ and the VFT-divergence temperature *T*_*VFT*_ with *ρ*_*pin*_ for the 3dKA and the 3dR10 models. For comparison all temperatures are scaled by the VFT-divergence temperature for the unpinned system, *T*_*VFT*0_. The lines join successive data points.

With these caveats, if the values of *T*_*C*_ and *T*_*VFT*_ extracted from the fits are used to construct a phase diagram in the (*ρ*_*pin*_ − *T*) plane, one finds somewhat puzzling results as depicted in Fig. 3. This figure shows the variation of *T*_*VFT*_ and *T*_*C*_, scaled by the value of *T*_*VFT*_ for *ρ*_*pin*_ = 0, with *ρ*_*pin*_. It is clear from the plots that *T*_*C*_ increases with increasing *ρ*_*pin*_, as predicted in Ref. 17 and in subsequent detailed MCT calculations^26^,^29^. On the other hand, *T*_*VFT*_ does not show any indication of increasing with *ρ*_*pin*_ to meet the *T*_*C*_ line at a critical value of *ρ*_*pin*_, as predicted in the MF analysis of Ref. 17. Rather, it seems to remain constant or to decrease slowly as *ρ*_*pin*_ is increased. To cross-check these results, we have obtained the parameters of the VFT form from so-called “Stickel plots”^30^. The Stickel analysis (described in the SI) is a method of obtaining the parameters appearing in the VFT equation from a linear fit of a particular derivative of the relaxation time data plotted against 1/*T*. We find that the values of *T*_*VFT*_ obtained from the lowest-temperature data points in the Stickel plots are close to those obtained from our VFT plots and exhibit very similar dependence on *ρ*_*pin*_. The Stickel plots do not show any indication that this behavior will change at lower temperatures.

**Figure 4:**
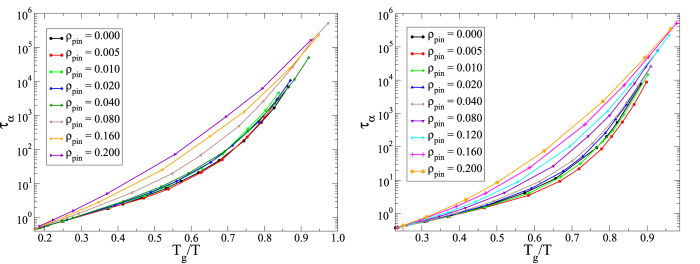
*Left panel:* **Angell plots** for the 3dKA model. *Right panel:* Angell plots for the 3dR10 model.

Another important and somewhat unexpected result of our study is that the kinetic fragility *K*_*VFT*_ decreases rapidly with increasing *ρ*_*pin*_ in both of our model systems. In the insets of the right panels of Figs 1 and 2, we have plotted *K*_*VFT*_ as a function of *ρ*_*pin*_. One can see that this measure of fragility changes by a factor of 5 − 8 in the studied range of *ρ*_*pin*_. To emphasize this point, we have constructed “Angell plots” in which the relaxation time is plotted as a function of the temperature scaled by *T*_*g*_, defined as *τ*_*α*_(*T*_*g*_) = 10^6^. These plots are also shown in Fig. 4. The dramatic change in the fragility, manifested as a change in the curvature of the plots, can be clearly seen in these Angell plots. Restricted fits in which *K*_*VFT*_ is fixed at the value for the unpinned liquid provide rather poor description of the data for relatively large values of *ρ*_*pin*_ (see the SI for details).

The phenomenological Adam-Gibbs relation^31^, *τ*_*α*_(*T*) ∝ exp[*B*/(*Ts*_*c*_(*T*))], between the *α*-relaxation time and the configurational entropy density (*B* is a constant) leads to a VFT form for the temperature dependence of *τ*_*α*_ with *T*_*VFT*_ = *T*_*K*_ if *s*_*c*_ behaves as *Ts*_*c*_(*T*) = *K*(*T* − *T*_*K*_) where *K* is a constant. Numerical results^32^,^33^ for the configurational entropy of liquids without pinning, obtained at relatively high temperatures at which the liquid can be equilibrated in time scales accessible in simulations, are consistent with this linear relation that extrapolates to zero at a temperature *T*_*K*_ that is close to the *T*_*VFT*_ obtained from a VFT fit to the temperature dependence of *τ*_*α*_. If we assume that the Adam-Gibbs relation remains valid^25^ in the presence of pinning and *s*_*c*_ actually goes to zero at *T* = *T*_*K*_ = *T*_*VFT*_ (as in the RFOT theory), then a reduction in *T*_*VFT*_ with increasing *ρ*_*pin*_ would not be consistent with the physically reasonable expectation that *s*_*c*_(*T*, *ρ*_*pin*_) is a decreasing function of *ρ*_*pin*_. However, our results are consistent, within error bars, with *T*_*VFT*_ being independent of *ρ*_*pin*_. This can be reconciled with the requirement of *s*_*c*_ decreasing with increasing *ρ*_*pin*_ if the dependence of *s*_*c*_ on *T* and *ρ*_*pin*_ is of the form


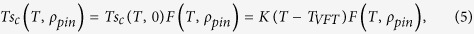


where *F*(*T*, *ρ*_*pin*_), the fractional reduction of the configurational entropy due to pinning, decreases from 1 as *ρ*_*pin*_ is increased from 0. If the temperature dependence of *F*(*T*, *ρ*_*pin*_) is weak (we ignore the dependence of *F* on *T* in the following discussion), then Eq. (5) and the Adam-Gibbs relation would lead to a VFT form for the temperature dependence of *τ*_*α*_, with *T*_*VFT*_ independent of *ρ*_*pin*_, in agreement with our observations. The fragility parameter *K*_*VFT*_ = *KT*_*VFT*_*F*(*ρ*_*pin*_)/*B*(*ρ*_*pin*_) would decrease with increasing *ρ*_*pin*_ (i.e. would agree with our observations) if *F*/B is a decreasing function of *ρ*_*pin*_. We already know that *F* is a decreasing function of *ρ*_*pin*_. The requirement that *F*/*B* be a decreasing function of *ρ*_*pin*_ would be satisfied if *B* increases, remains constant or decreases slower than *F* with increasing *ρ*_*pin*_. In this scenario, the temperature dependence of *τ*_*α*_ for non-zero *ρ*_*pin*_ is given by


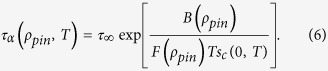


This implies the following relation between the relaxation times with and without pinning:


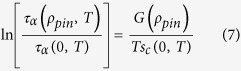


where the function *G*(*ρ*_*pin*_) is defined by


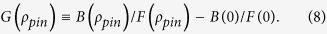


It is clear from the definition that *G*(0) = 0 and assuming that *G* and *F* are smooth functions of *ρ*_*pin*_, we get


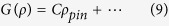


where *C* is a constant and 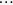 represent terms with higher powers of *ρ*_*pin*_, which can be neglected for small values of *ρ*_*pin*_. So, for small values of *ρ*_*pin*_, Eq. (7) becomes


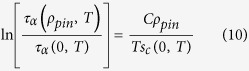


which has the form of a scaling relation,


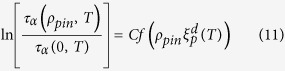


with *f*(*x*) = *x* and the pinning length scale *ξ*_*p*_ given by *ξ*_*p*_(*T*) = [1/(*Ts*_*c*_(0,*T*))]^1/*d*^ ∝ [1/(*T* − *T*_*VFT*_)]^1/*d*^ (*d* is the spatial dimension).

**Figure 5:**
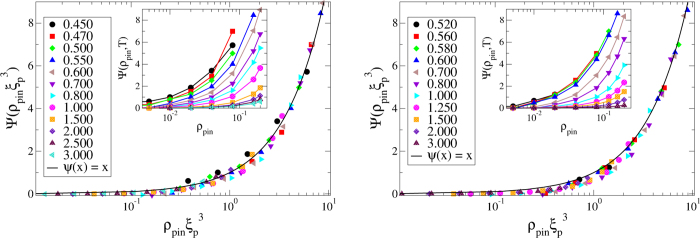
Validation of Scaling Ansatz. Data collapse using the scaling form in Eq. (11) to obtain the length-scale *ξ*_*p*_. Left panel is for the 3dKA system and the right panel is for the 3dR10 system. In both cases, insets show the uncollapsed data.

**Figure 6:**
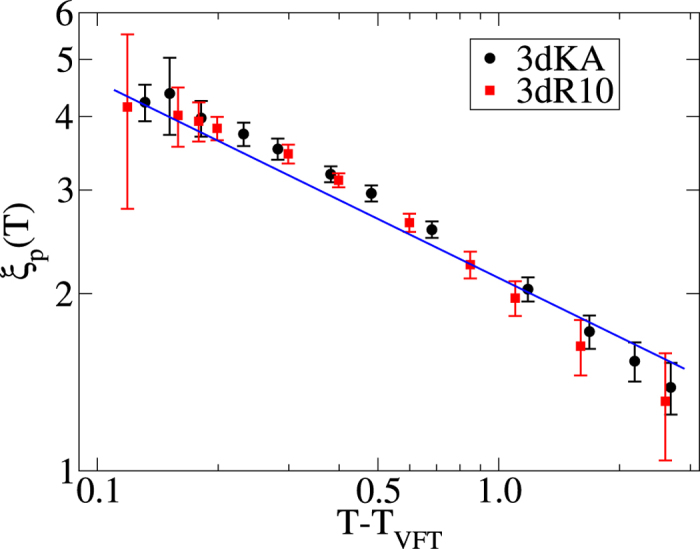
Pinning length scale *ξ*_*p*_ as a function of temperature *T*. The straight line has a slope of −1/3.

To test this scaling prediction, we have tried to collapse the data for *ψ*(*ρ*_*pin*_, *T*) ≡ ln[*τ*_*α*_(*ρ*_*pin*_,*T*)/*τ*_*α*_(0, *T*)] for all temperatures and pinning densities into a single scaling curve by re-scaling the *ρ*_*pin*_-axis of the data for each temperature by a factor 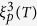. The length scales *ξ*_*p*_(*T*) at different temperatures are treated as adjustable parameters whose values are chosen to obtain the best collapse of the *ψ*(*ρ*_*pin*_, *T*) *versus* 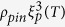 plots for all *T* and *ρ*_*pin*_ to a single master curve. As shown in Fig. 5, good data collapse is obtained for both the model systems studied. The line passing through the collapsed data is indeed of the form predicted by the scaling argument. We have also checked whether the *ξ*_*p*_(*T*) obtained from the scaling collapse is proportional to [1/(*T* − *T*_*VFT*_)]^1/3^. In Fig. 6, we have plotted the pinning length scale *ξ*_*p*_ as a function of (*T* − *T*_*VFT*_) for both the model systems. The results are clearly consistent with the scaling argument. It is interesting to note that although our results for the phase diagram do not agree with the prediction of Ref. 17, the temperature dependence of the pinning length *ξ*_*p*_(*T*) turns out to be the same as that in Ref. 17. Preliminary results of a study^34^ of two-dimensional pinned liquids suggest that the exponent that describes the temperature dependence of *ξ*_*p*_ is 1/2 in two dimensions.

It is also possible that *F*(*ρ*_*pin*_) goes to zero at a value of *ρ*_*pin*_ higher than the largest value considered in our simulations. If this happens, then the line of (putative) thermodynamic glass transitions in the (*T* − *ρ*_*pin*_) plane would end at this “critical” value of *ρ*_*pin*_. This would be similar to the phase diagram obtained in the RG calculation reported in Ref. 17, with the important difference that the transition line would be parallel to the *ρ*_*pin*_ axis. It should, however, be noted that the arguments above do not depend on the assumption that a thermodynamic glass transition arising from the vanishing of *s*_*c*_ actually occurs in liquids without pinning. Since numerically obtained values of *s*_*c*_(*T*, 0) at the relatively high temperatures considered in our simulations satisfy the relation *Ts*_*c*_(*T*, 0) = *K*(*T* − *T*_*VFT*_) irrespective of whether *s*_*c*_ actually goes to zero at a non-zero temperature, our results are also compatible with scenarios, such as those based on the behavior of kinetically constrained models^18^, in which a thermodynamic glass transition does not occur at any non-zero temperature. In such a scenario, a slow reduction of the value of *T*_*VFT*_, obtained from fitting the simulation data for *τ*_*α*_ to the VFT form, with increasing pin concentration would not be incompatible with the physical requirement of *s*_*c*_ decreasing with increasing *ρ*_*pin*_.

### Comparison with Other Numerical Results

Our results for the phase diagram in the (*ρ*_*pin*_ − *T*) plane, with *T*_*VFT*_ identified with *T*_*K*_, are in disagreement with those of two recent numerical studies^24^,^35^. In Ref. 24, the dependence of *T*_*K*_ on *ρ*_*pin*_ was obtained from simulations of a 64-particle system of harmonic spheres. The temperature *T*_*K*_, obtained from the behavior of the distribution of an overlap parameter similar to that defined in Eq. (4), was found to increase with increasing *ρ*_*pin*_. We believe that the results reported in Ref. 24 suffer from strong finite-size effects. As shown in that paper, the basic form of the distribution (whether it is unimodal or bimodal) for a system with 64 particles can be different from that for a system with 128 particles. Similar results for strong finite-size effects in the distribution of a similar overlap parameter were reported earlier in Ref. 12 which provides a detailed account of finite-size effects in the equilibrium and dynamic properties of the 3dKA model. Since a change in the distribution from unimodal to bimodal is supposed to signal the transition from the liquid to the glass phase, the data for the distribution obtained for a system with 64 particles cannot provide reliable quantitative information about the location of the transition point in the (*ρ*_*pin*_ − *T*) plane. This, we believe, is the primary reason for the difference between our results and those of Ref. 24. We have also found that the fragility for a 64-particle system (3dR10 model) is substantially smaller than that for a system with 1000 particles. Since our results show that the main effect of pinning is a reduction of the fragility, studies of small systems in which the fragility is strongly affected by system size are not expected to provide a reliable description of the effects of pinning.

In another recent study^35^, the configurational entropy density *s*_*c*_ of the 3dKA model was calculated for different pin densities and temperatures and the phase boundary in the (*ρ*_*pin*_ − *T*) plane was obtained by estimating the values of *ρ*_*pin*_ at which *s*_*c*_ goes to zero for different temperatures. The value of *T*_*K*_ obtained in this way was found to increase with increasing pin concentration. These results, obtained from the behavior of thermodynamic quantities, are quite different from those obtained in our work from the dynamics of the system.

**Figure 7:**
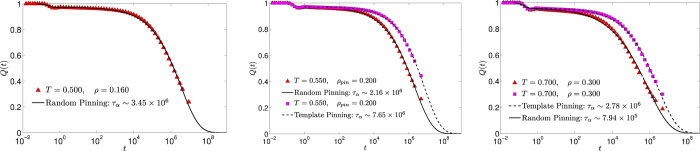
Overlap correlation function *Q*(*t*) for three different state points, (*T* = 0.500, *ρ*_*pin*_ = 0.160), (*T* = 0.550, *ρ*_*pin*_ = 0.200) and (*T* = 0.700, *ρ*_*pin*_ = 0.300), near the phase boundary obtained in Ref. 35 from vanishing of the configurational entropy. The system size, *N* = 300, is the same as that in Ref. 35. Results for *Q*(*t*) obtained using the template protocol for selecting the pinned particles are shown in the middle and right panels for comparison. The lines are fit of the *Q*(*t*) data using the functional form 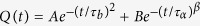 with *A*, *B*, *τ*_*b*_, *τ*_*α*_, *β* as variables.

To resolve this discrepancy, we have simulated the dynamics of the 3dKA model at a few points in the (*ρ*_*pin*_ − *T*) plane at which *s*_*c*_ is supposed to go to zero according to the phase diagram obtained in Ref. 35. In these simulations, we considered the same system size (*N* = 300) as that in Ref. 35 to remove any ambiguity that may arise from finite-size effects (we found similar results for *N* = 1000, indicating that finite-size effects for the dynamics are weak for systems with 300 or more particles). Surprisingly, we found that it is possible to equilibrate the system in time scales accessible in MD simulations at the points where *s*_*c*_ is supposed to go to zero or to have a very small value. Results for the overlap function *Q*(*t*) at three “transition points”, (*T* = 0.50, *ρ*_*pin*_ = 0.16), (*T* = 0.55, *ρ*_*pin*_ = 0.20) and (*T* = 0.70, *ρ*_*pin*_ = 0.30), are shown in Fig. 7. The relaxation times at these points are estimated to be of the order of 10^6^ − 10^7^, as indicated in the legends of the respective plots. This is very different from the behavior of systems without pinning, for which it is impossible to equilibrate the system in time scales accessible in MD simulations at temperatures close to the value at which *s*_*c*_ is supposed to go to zero. For example, *τ*_*α*_ in the unpinned 3dKA model attains the value of 10^7^ at a temperature that is 25% higher than the temperature at which *s*_*c*_ extrapolates to zero.

These results seem to imply that the relaxation time is *finite* at points in the (*ρ*_*pin*_ − *T*) plane where *s*_*c*_ goes to zero (or has a very small value) according to the results reported in Ref. 35. While the possibility that the relaxation time does not diverge when the configurational entropy vanishes cannot be ruled out, we believe that this is unlikely to be the explanation of the seemingly contradictory results mentioned above. The dependence of *τ*_*α*_ on *s*_*c*_ may not be the Adam-Gibbs relation (it is suggested in Ref. 35 that the Adam-Gibbs relation is violated in pinned systems), but a violation of the physically reasonable expectation that *τ*_*α*_ should diverge if *s*_*c*_ goes to zero would be quite surprising. A more probable explanation of these results is that *s*_*c*_ does not actually go to zero at the phase boundary obtained in Ref. 35. The calculation of *s*_*c*_ in Ref. 35 requires estimation of the “basin entropy” which is calculated using a harmonic approximation. Anharmonic effects at the relatively high temperatures considered for pinned systems may cause inaccuracies in the estimation of *s*_*c*_, leading to errors in the determination of the phase boundary in the (*ρ*_*pin*_ − *T*) plane. One of the signatures of this effect has been pointed out by the authors of Ref. 35 as the negative values of the configurational entropy at some state points. Another point to note is that the pinned particles were chosen randomly in our simulations, whereas a “template” method was used in Ref. 35. We have found that time scales obtained for template pinning are systematically larger than those for randomly pinned systems at the same temperature and pin density. Typical results for (*T* = 0.55, *ρ*_*pin*_ = 0.20) and (*T* = 0.70, *ρ*_*pin*_ = 0.30) are shown in the middle and right panels of Fig. 7. It is not clear whether this difference between the protocols used for selecting the pinned particles would account for the difference between our results and those of Ref. 35. It would be interesting to find out whether the configurational entropy is affected by the choice of the protocol. It is argued in Ref. 35 that template pinning reduces sample-to-sample fluctuations compared to random pinning, but we found that fluctuations in the overlap function are of similar magnitude for both protocols. It is also possible (but very unlikely) that the plots for *Q*(*t*) in Fig. 7 would level off at time scales longer than those considered in our simulations, leading to very large values of the relaxation time. The excellent fits of the data for *Q*(*t*) to a stretched exponential form, shown in Fig. 7, strongly argues against this possibility.

In a recent numerical study^36^ of the dynamics of the Kob-Andersen mixture in the presence of random pinning of the kind considered here, it was found, from VFT fits to the data for *τ*_*α*_ as a function of *ρ*_*pin*_ at a fixed temperature *T*, that the values of *ρ*_*pin*_ at which *τ*_*α*_ appears to diverge are large (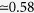) and essentially independent of *T* for *T* ≤ 0.7. These “critical” values of *ρ*_*pin*_ are very different from those predicted in the phase diagram of Ref. 35. The dynamic behavior found in Ref. 36 is qualitatively similar to that found in our study and consistent with the behavior of the configurational entropy proposed in Eq. (5) with *F*(*ρ*_*pin*_) going to zero at 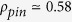.

## Conclusion

In summary, we have carried out a detailed numerical study of the dynamics of two model glass forming liquids with randomly pinned particles. Using standard methods to analyze the observed temperature dependence of the structural relaxation time *τ*_*α*_, we find that the MCT temperature *T*_*C*_ increases and the VFT-divergence temperature *T*_*VFT*_ remains nearly constant or decreases slowly with increasing pin concentration. The observed dependence of *T*_*C*_ on *ρ*_*pin*_ is consistent with the prediction of Ref. 17, but the dependence of *T*_*VFT*_ on *ρ*_*pin*_ is in disagreement with the theoretical prediction of Ref. 17 and the numerical results reported in Refs 24 and 35 if we assume (as is done in the RFOT description) that a thermodynamic transition at which the configurational entropy density *s*_*c*_ goes to zero coincides with a divergence of *τ*_*α*_. We have also found that the system can be equilibrated in time scales accessible in MD simulations at state points in the (*ρ*_*pin*_ − *T*) plane at which *s*_*c*_ is supposed to vanish according to the phase diagram of Ref. 35. Since the values of *T*_*VFT*_ are obtained in our work from extrapolations based on the VFT form, one can argue that the discrepancy between the *T*_*K*_ values obtained in Ref. 35 and the values of *T*_*VFT*_ obtained in our study is caused by errors introduced by extrapolation. However, this argument can not explain our observation of *τ*_*α*_ being finite (and not very large) at state points where *s*_*c*_ is zero or very small according to the results of Ref. 35. This observation allows only two alternative interpretations. The first interpretation is that the *α*-relaxation time in randomly pinned liquids does not diverge as the configurational entropy density goes to zero. This interpretation would imply that a causal relation between the vanishing of *s*_*c*_ and the divergence of *τ*_*α*_, the existence of which is the basic premise of RFOT, is not present in randomly pinned liquids. This would also imply that the entropy vanishing “transition” found in Ref. 35 should not be called a glass transition because the divergence of the relaxation time is a defining feature of the glass transition. The second interpretation is that the numerical results for *s*_*c*_ obtained in Ref. 35 are not quantitatively correct - *s*_*c*_ does not actually go to zero at the phase boundary obtained in this work. In our opinion, this interpretation is more likely to be correct - anharmonic effects in the calculation of the basin entropy may introduce errors in the numerical estimation of *s*_*c*_.

If we disregard the results for *s*_*c*_ reported in Ref. 35, then a complete understanding of our results for the dynamics can be obtained from a description that is consistent with RFOT and the physical requirement that the value of *s*_*c*_ must decrease with increasing pin concentration. In this description, the configurational entropy density of a system with pinning is assumed to be obtained by multiplying the value of *s*_*c*_ for the unpinned system at the same temperature by a factor that is less than unity, essentially independent of *T* and a decreasing function of the pin concentration. As discussed in the “Results” section, this description provides a very good understanding of all the features observed in our simulations of the dynamics of two pinned liquids. It also provides a qualitative understanding of the numerical results reported in Ref. 36. In this description, the phase diagram in the (*ρ*_*pin*_ − *T*) plane is similar to that obtained in the RG calculation of Ref. 17, but with the important difference that the transition line is parallel to the *ρ*_*pin*_ axis.

These findings will help in interpreting the results of experiments^20^ on colloidal systems with random pinning. We also find a rapid reduction of the kinetic fragility with increasing pin concentration. Since the fragility changes by factor of five to eight as the pin concentration is changed, model liquids with randomly pinned particles may also be useful for understanding the role of fragility in the glass transition.

## Additional Information

**How to cite this article**: Chakrabarty, S. *et al.* Dynamics of Glass Forming Liquids with Randomly Pinned Particles. *Sci. Rep.* **5**, 12577; doi: 10.1038/srep12577 (2015).

## Supplementary Material

Supplementary Information

## References

[b1] Cavagna A. Supercooled liquids for pedestrians. Phys. Rep. 476, 51–124 (2009).

[b2] Berthier L. & Biroli G. Theoretical perspective on the glass transition and amorphous materials. Rev. Mod. Phys. 83, 587–645 (2011).

[b3] Kirkpatrick T. R., Thirumalai D. & Wolynes P. G. Scaling concepts for the dynamics of viscous liquids near an ideal glassy state. Phys. Rev. A 40, 1045–1054 (1989). 9902230 10.1103/physreva.40.1045

[b4] Lubchenko V. & Wolynes P. G. Theory of structural glasses and supercooled liquids. Annu. Rev. Phys. Chem. 58, 235–266 (2007). 17067282 10.1146/annurev.physchem.58.032806.104653

[b5] Biroli G. & Bouchaud J.-P. The random first-order transition theory of glasses: a critical assessment. Structural Glasses and Supercooled Liquids: Theory, Experiment, and Applications 31–113 (2012).

[b6] Chandler D. & Garrahan J. P. Dynamics on the way to forming glass: Bubbles in space-time. Ann. Rev. Phys. Chem. 61, 191–217 (2007). 20055676 10.1146/annurev.physchem.040808.090405

[b7] Karmakar S., Dasgupta C. & Sastry S. Growing length scales and their relation to timescales in glass-forming liquids. Annu. Rev. Condens. Matter Phys. 5, 255–284 (2014).

[b8] Ediger M. D. Spatially heterogeneous dynamics in supercooled liquids. Annu. Rev. Phys. Chem. 51, 99–128 (2000). 11031277 10.1146/annurev.physchem.51.1.99

[b9] Berthier L. *et al.* Direct experimental evidence of a growing length scale accompanying the glass transition. Science 310, 1797–1800 (2005). 16357256 10.1126/science.1120714

[b10] Biroli G., Bouchaud J.-P., Miyazaki K. & Reichman D. R. Inhomogeneous mode-coupling theory and growing dynamic length in supercooled liquids. Phys. Rev. Lett. 97, 195701 (2006). 17155642 10.1103/PhysRevLett.97.195701

[b11] Biroli G., Bouchaud J.-P., Cavagna A., Grigera T. S. & Verrocchio P. Thermodynamic signature of growing amorphous order in glass-forming liquids. Nat. Phys. 4, 771–775 (2008).

[b12] Karmakar S., Dasgupta C. & Sastry S. Growing length and time scales in glass-forming liquids. Proc. Nat’l Acad. Sci. USA 106, 3675–3679 (2009). 19234111 10.1073/pnas.0811082106 PMC2656139

[b13] Karmakar S., Lerner E. & Procaccia I. Direct estimate of the static length-scale accompanying the glass transition. Physica A: Statistical Mechanics and its Applications 391, 1001–1008 (2012).

[b14] Hocky G. M., Markland T. E. & Reichman D. R. Growing point-to-set length scale correlates with growing relaxation times in model supercooled liquids. Phys. Rev. Lett. 108, 225506 (2012). 23003622 10.1103/PhysRevLett.108.225506

[b15] Karmakar S. & Procaccia I. Finite-size scaling for the glass transition: The role of a static length scale. Phys. Rev. E 86, 061502 (2012). 10.1103/PhysRevE.86.061502 23367953

[b16] Biroli G., Karmakar S. & Procaccia I. Comparison of static length scales characterizing the glass transition. Phys. Rev. Lett. 111, 165701 (2013). 24182280 10.1103/PhysRevLett.111.165701

[b17] Cammarota C. & Biroli G. Ideal glass transitions by random pinning. Proc. Nat’l Acad. Sci. USA 109, 8850–8855 (2012). 22623524 10.1073/pnas.1111582109 PMC3384138

[b18] Garrahan J. P., Sollich P. & Toninelli C. Kinetically constrained models. Dynamical heterogeneities in Glasses, colloids and granular media and jamming transitions, International series of monographs in physics (Oxford University Press, Oxford, 2011) Chap 10, 341–369 (2011).

[b19] Jack R. L. & Berthier L. Random pinning in glassy spin models with plaquette interactions. Phys. Rev. E 85, 021120 (2012). 10.1103/PhysRevE.85.021120 22463166

[b20] Gokhale S., Nagamanasa K. H., Ganapathy R. & Sood A. Growing dynamical facilitation on approaching the random pinning colloidal glass transition. Nature communications 5 (2014). 10.1038/ncomms5685 25119444

[b21] Karmakar S. & Parisi G. Random pinning glass model. Proc. Nat’l Acad. Sci. USA 110, 2752–2757 (2013). 23382186 10.1073/pnas.1222848110 PMC3581890

[b22] Kim K. Effects of pinned particles on the structural relaxation of supercooled liquids. Europhys. Lett. 61, 790 (2003).

[b23] Berthier L. & Kob W. Static point-to-set correlations in glass-forming liquids. Phys. Rev. E 85, 011102 (2012). 10.1103/PhysRevE.85.011102 22400507

[b24] Kob W. & Berthier L. Probing a liquid to glass transition in equilibrium. Phys. Rev. Lett. 110, 245702 (2013). 25165938 10.1103/PhysRevLett.110.245702

[b25] Kob W. & Coslovich D. Nonlinear dynamic response of glass-forming liquids to random pinning. Phys. Rev. E 90, 052305 (2014). 10.1103/PhysRevE.90.052305 25493794

[b26] Krakoviack V. Mode-coupling theory predictions for the dynamical transitions of partly pinned fluid systems. Phys. Rev. E 84, 050501 (2011). 10.1103/PhysRevE.84.050501 22181359

[b27] Das S. P. Mode-coupling theory and the glass transition in supercooled liquids. Reviews of modern physics 76, 785 (2004).

[b28] Kob W. & Andersen H. C. Testing mode-coupling theory for a supercooled binary Lennard-Jones mixture I: The van Hove correlation function. Phys. Rev. E 51, 4626–4641 (1995). 10.1103/physreve.51.4626 9963176

[b29] Szamel G. & Flenner E. Glassy dynamics of partially pinned fluids: An alternative mode-coupling approach. Europhys. Lett. 101, 66005 (2013).

[b30] Stickel F., Fischer E. & Richert R. Dynamics of glass-forming liquids. i. temperature-derivative analysis of dielectric relaxation data. J. Chem. Phys. 102, 6251–6257 (1995).

[b31] Adam G. & Gibbs J. H. On the temperature dependence of cooperative relaxation properties in glass-forming liquids. J. Chem. Phys. 43, 139–146 (1965).

[b32] Sengupta S., Karmakar S., Dasgupta C. & Sastry S. Adam-gibbs relation for glass-forming liquids in two, three, and four dimensions. Phys. Rev. Lett. 109, 095705 (2012). 23002857 10.1103/PhysRevLett.109.095705

[b33] Sengupta S., Vasconcelos F., Affouard F. & Sastry S. Dependence of the fragility of a glass former on the softness of interparticle interactions. J. Chem. Phys. 135, 194503 (2011). 22112088 10.1063/1.3660201

[b34] Chakrabarty S., Das R., Karmakar S. & Dasgupta C. manuscript in preparation (2015).

[b35] Ozawa M., Kob W., Ikeda A. & Miyazaki K. Equilibrium phase diagram of a randomly pinned glass-former. *arXiv:1412.4911* (2014). 10.1073/pnas.1500730112 PMC4460508 25976100

[b36] Li Yan-Wei, Zhu You-Liang & Sun Zhao-Yan Decoupling of relaxation and diffusion in random pinning glass-forming liquids. J. Chem. Phys. 142, 124507 (2015). 25833596 10.1063/1.4916208

