# Supplementary material for: Dynamics of Glass Forming Liquids with Randomly Pinned Particles
Source: Sci Rep. 2015 Jul 24;5:12577. doi: 10.1038/srep12577 (PMC4513344; doi:10.1038/srep12577)
Supplement: Supplementary Information [file srep12577-s1.pdf]

Dynamics of Glass Forming Liquids with Randomly Pinned Particles – Supplementary Information

Saurish Chakrabarty[1], Smarajit Karmakar[2], and Chandan Dasgupta[1,3]
[1] *Centre for Condensed Matter Theory, Department of Physics, Indian Institute of Science, Bangalore, 560012, India,*
[2] *Centre for Interdisciplinary Sciences, Tata Institute of Fundamental Research, 21 Brundavan Colony, Narisingi, Hyderabad, India,*
[3] *Jawaharlal Nehru Centre for Advanced Scientific Research, Bangalore 560064, India.*

I. MODEL AND SIMULATION

The first model glass former we study is the well-known Kob-Andersen [1] 80 : 20 binary Lenard-Jones mixture. In the main article it is referred to as the 3dKA model. The interaction potential in this model is given by

$$V_{\alpha\beta}(r) = 4\epsilon_{\alpha\beta}\left[\left(\frac{\sigma_{\alpha\beta}}{r}\right)^{12} - \left(\frac{\sigma_{\alpha\beta}}{r}\right)^{6}\right], \qquad (1)$$

where $\alpha, \beta \in \{A, B\}$ and $\epsilon_{AA} = 1.0$, $\epsilon_{AB} = 1.5$, $\epsilon_{BB} = 0.5$, $\sigma_{AA} = 1.0$, $\sigma_{AB} = 0.80$, $\sigma_{BB} = 0.88$. The interaction potential is cut off at $2.50\sigma_{\alpha\beta}$ and we use a quadratic polynomial to make the potential and its first two derivatives smooth at the cutoff distance. The temperature range studied for this model is $T \in \{0.450, 3.000\}$ at number density $\rho = 1.20$. The second model studied is a 50 : 50 binary mixture with pairwise interactions between the particles that falls off with distance as an inverse power law as

$$V_{\alpha\beta}(r) = \epsilon_{\alpha\beta}\left(\frac{\sigma_{\alpha\beta}}{r}\right)^{n}, \qquad (2)$$

with exponent $n = 10$ (the 3dR10 model). The potential is cut off at $1.38\sigma_{\alpha\beta}$. We again use a quadratic polynomial to make the potential and its first two derivative smooth at the cutoff. The parameters of the potential are: $\epsilon_{\alpha\beta} = 1.0$, $\sigma_{AA} = 1.0$, $\sigma_{AB} = 1.22$ and $\sigma_{BB} = 1.40$. The temperature range covered for this model is $T \in \{0.52, 3.00\}$ at number density $\rho = 0.81$.

NVT molecular dynamics simulations are done in a cubic simulation box with periodic boundary conditions in three dimensions for both the model systems. We use the modified leap-frog algorithm with the Berendsen thermostat to keep the temperature constant in the simulation runs. Length, energy and time scales are measured in units of σ_{AA}, ϵ_{AA} and $\sqrt{\sigma_{AA}^2/\epsilon_{AA}}$. The integration time step used is $dt = 0.005$ in this temperature range. Equilibration runs are performed for $\sim 10^8 - 10^9$ MD steps depending on the temperature and production runs are long enough to ensure that the two-point density correlation function $Q(t)$ goes to zero within the simulation time. For both the model systems, we have performed simulations for pin concentration in the range $\rho_{pin} \in \{0.005, 0.200\}$ for each temperature. For very low temperatures, we were not able to equilibrate the system for high pin concentrations because of a dramatic increase in the relaxation time in these cases.

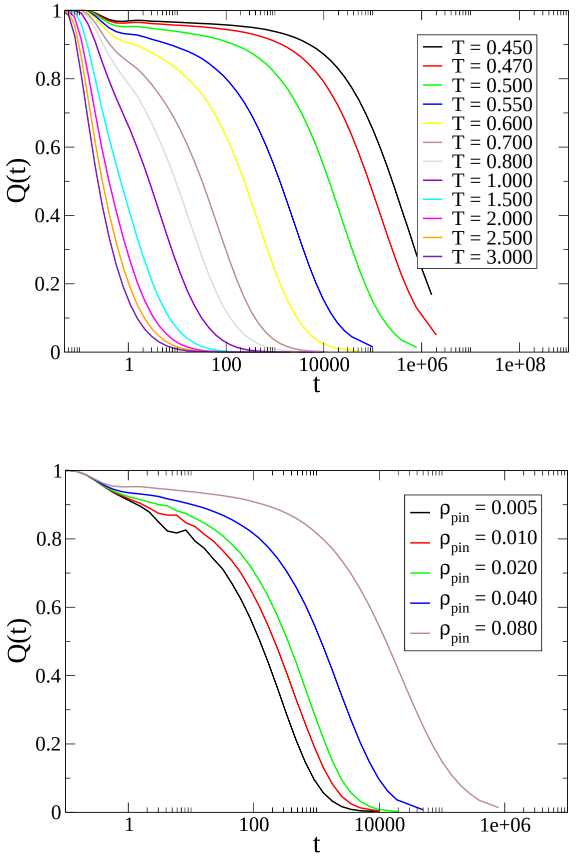

FIG. 1: *Top Panel:* $Q(t)$ for $\rho_{pin} = 0.080$ for various temperatures for the $3dKA$ model. *Bottom Panel:* $Q(t)$ for $T = 0.500$ for various levels of pinning for the $3dKA$ model.

II. TYPICAL OVERLAP CORRELATION FUNCTIONS

Some typical overlap correlation functions $Q(t)$, from our simulations have been plotted in Figs. 1 and 2. The set has data covering the entire range of relaxation times we obtained. The relaxation time τ_α was calculated using $Q(\tau_\alpha) = \exp(-1)$.

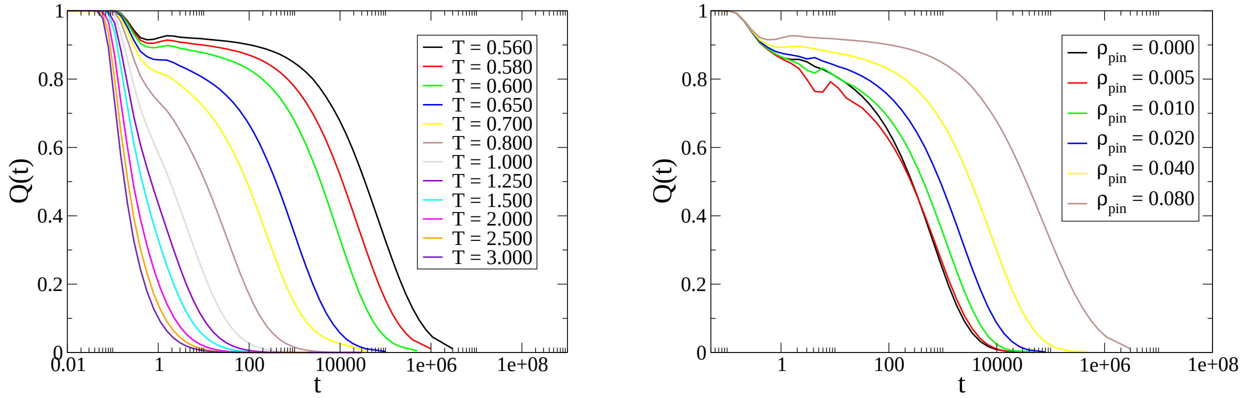

FIG. 2: *Left Panel:* $Q(t)$ for $\rho_{pin} = 0.080$ for various temperatures for the $3dR10$ model. *Right Panel:* $Q(t)$ for $T = 0.560$ for various levels of pinning for the $3dR10$ model.

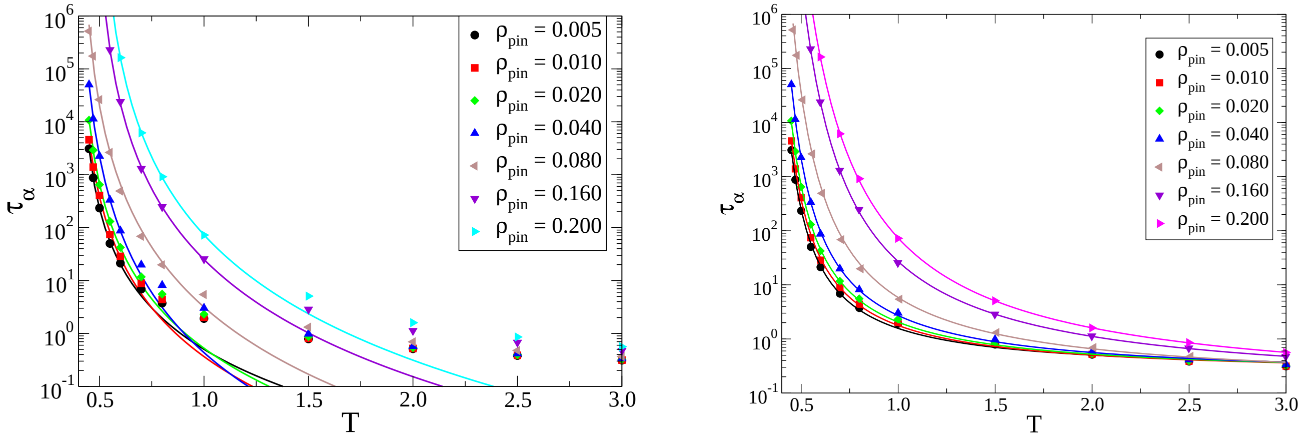

FIG. 3: *Left Panel:* Power law fit to obtain T_C as a function of ρ_{pin} for the $3dKA$ model. *Right Panel:* VFT fit to obtain T_{VFT} as a function of ρ_{pin} for the $3dKA$ model.

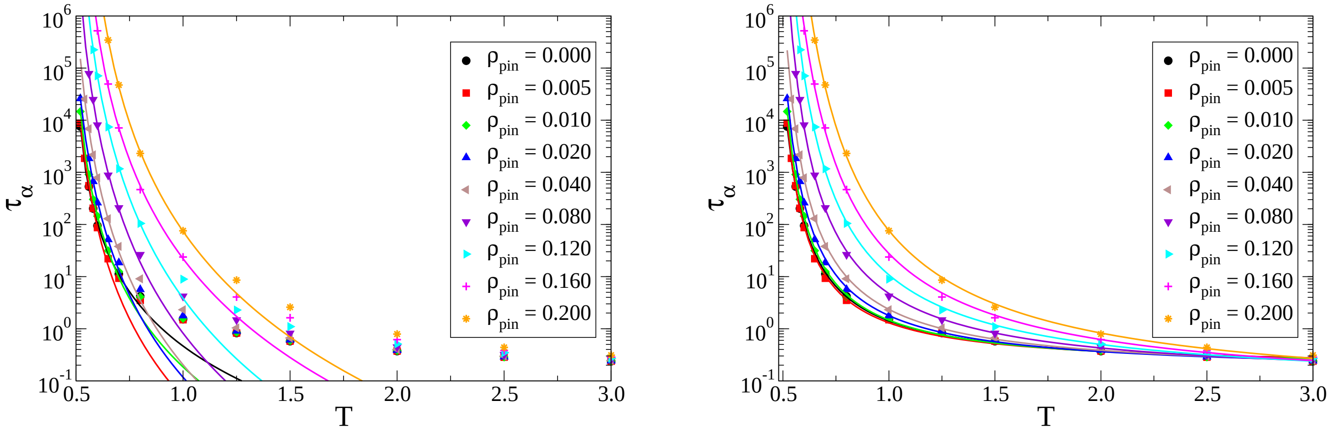

FIG. 4: *Left Panel:* Power law fit to obtain T_C as a function of ρ_{pin} for the $3dR10$ model. *Right Panel:* VFT fit to obtain T_{VFT} as a function of ρ_{pin} for the $3dR10$ model.

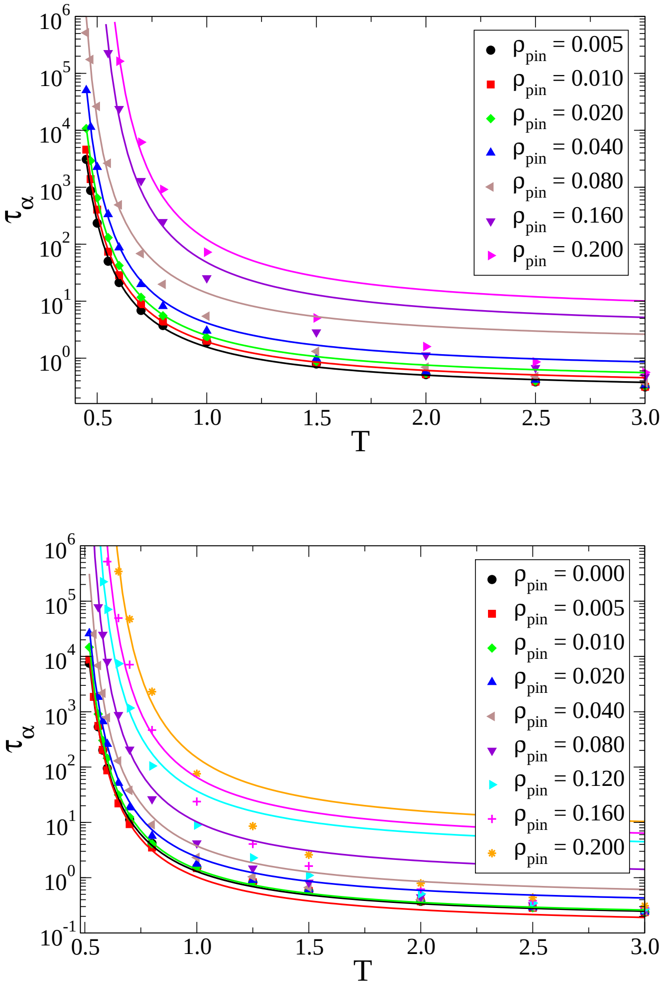

FIG. 5: *Left Panel:* VFT fit with fixed fragility for the $3dKA$ model. *Right Panel:* VFT fit with fixed fragility for the $3dR10$ model.

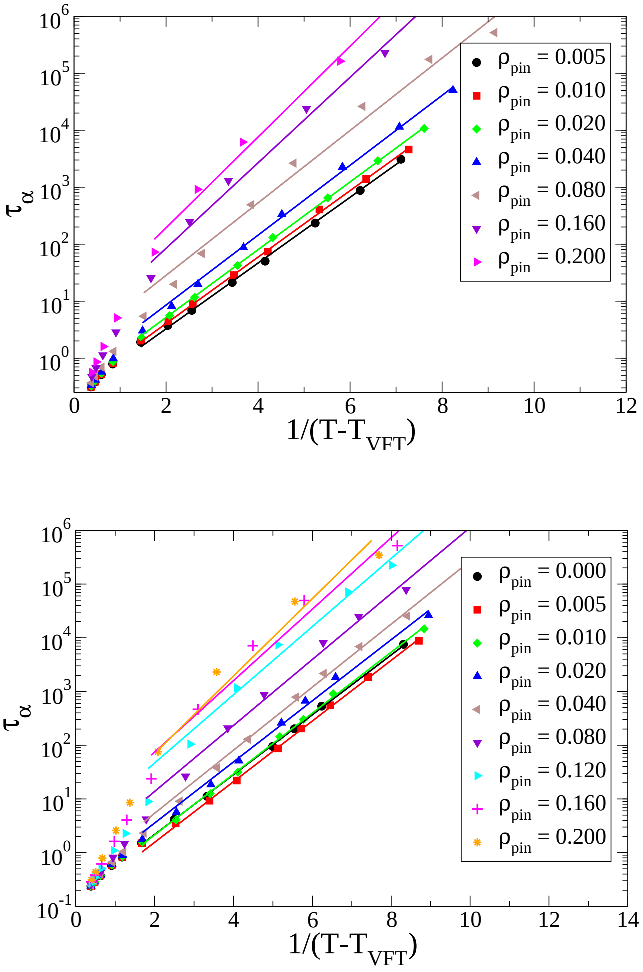

FIG. 6: *Left Panel:* Straight line plots for the VFT fit with fixed fragility for the $3dKA$ model. *Right Panel:* Straight line plots for the VFT fit with fixed fragility for the $3dR10$ model.

III. FITTING PROCEDURE

The fitting was done using the method of least squares. The natural logarithm of the relaxation time, $\ln(\tau_\alpha)$ was calculated first and the fitting was done using the $\ln(\tau_\alpha)$ versus T data. The error bars for the parameters obtained from the fit were calculated from the error in the τ_α-data using the following procedure. For each value of τ_α, we generated uniformly distributed data lying within the full width at half maximum of a Gaussian distribution with mean τ_α and variance $(\delta\tau_\alpha)^2$ ($\delta\tau_\alpha$ being the error in τ_α). Fits were done to 100 independent sets of such data. The spread in the parameters obtained from these fits were taken as the errors in the fit parameters.

The power-law and VFT fits for the two studied models are shown in Figs. 3 and 4. The same has been presented in the main paper in a way so that the fits appear as straight lines. The T_{VFT} and T_C values obtained from the fits are plotted in Tables I and II.

ρ_{pin}	T_{VFT}	T_C
0.000	0.4012±0.0102	0.4852±0.0165
0.005	0.4122±0.0126	0.4567±0.0396
0.010	0.4052±0.0152	0.4721±0.0352
0.020	0.3942±0.0092	0.4492±0.0324
0.040	0.3992±0.0099	0.4595±0.0401
0.080	0.3872±0.0246	0.4643±0.0447
0.120	0.3722±0.0225	0.4740±0.0429
0.160	0.3502±0.0242	0.4996±0.0499
0.200	0.3432±0.0289	0.5171±0.0365

TABLE I: T_{VFT} and T_C values obtained from the VFT and MCT fits at various levels of pinning for the 3dR10 model.

ρ_{pin}	T_{VFT}	T_C
0.005	0.3092±0.0098	0.4092±0.0163
0.010	0.2993±0.0092	0.3921±0.0310
0.020	0.2993±0.0112	0.4010±0.0300
0.040	0.2993±0.0095	0.3935±0.0157
0.080	0.2843±0.0173	0.4039±0.0208
0.160	0.2733±0.0349	0.4712±0.0377
0.200	0.2494±0.0528	0.4899±0.0270

TABLE II: T_{VFT} and T_C values obtained from the VFT and MCT fits at various levels of pinning for the 3dKA model.

A. Power law divergence

The form assumed was

$$\tau_\alpha = \frac{A}{(T - T_C)^\gamma}, \tag{3}$$

where A is a constant pre-factor having appropriate dimensions, γ is the exponent for the divergence, and T_C is the (putative) transition temperature of mode coupling theory. For each value of the pinning density ρ_{pin}, 4-5 data points with the highest values of τ_α were used for the fitting procedure.

B. VFT divergence

The form assumed was

$$\tau_\alpha = \tau_\infty \exp\left[\frac{1}{K_{VFT}\left(\frac{T}{T_{VFT}} - 1\right)}\right], \tag{4}$$

where τ_∞ is the high temperature relaxation time, K_{VFT} is the kinetic fragility and T_{VFT} is the Kauzmann temperature.

1. *Fixed fragility*

We obtained the same VFT fits also with fixed fragility. These are shown in Figs. 5 and 6. Since the fragility does change with pinning density, these fits were good only in the low temperature regime. As such, for these fits, we only used those data points for which $\tau_\alpha > 10$. The phase diagram obtained using these fits is shown in the left panel of Fig. 7.

C. Onset temperature

The onset of super-Arrhenius behavior is marked by the temperature T_{onset}. This has been estimated by observing the meeting of the Arrhenius fit to the high temperature data and the super-Arrhenius fit to the low temperature data. This meeting point marks the crossover

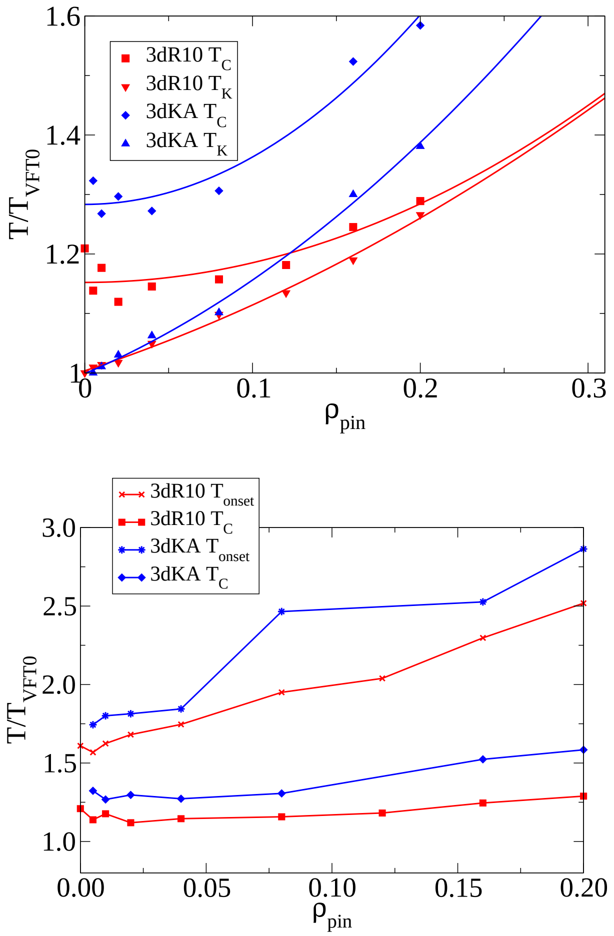

FIG. 7: *Left Panel:* The phase diagram with values of T_{VFT} obtained from fits with a fixed value of the kinetic fragility. The lines are quadratic fits to the data. The fits for the T_C data were done without a linear term. *Right Panel:* Variation of the MCT-transition temperature T_C and the onset temperature T_{onset} with ρ_{pin} for the 3dKA and the 3dR10 models.

from the high temperature Arrhenius behavior to the low temperature super-Arrhenius behavior. The predictions based on MF and RFOT analysis suggest that T_{onset} should approach T_C as ρ_{pin} is increased [2]. Our results, plotted in the right panel of Fig. 7, do not support this prediction.

D. Stickel Plots

An efficient way to verify the validity of the VFT form for the divergence of the relaxation time at T_{VFT} was introduced in Ref. [3]. This method involves analyses of the derivative of the viscosity (or α relaxation time) with respect to the temperature. A quantity $\Phi(T)$ is cal-

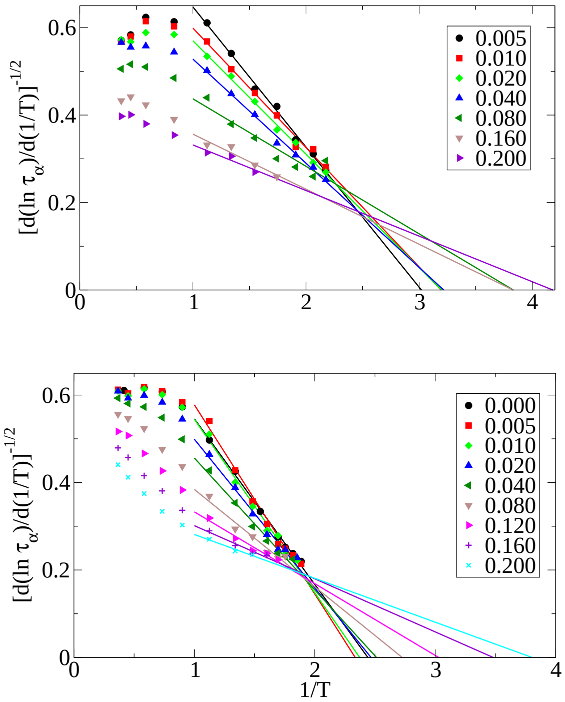

FIG. 8: Inverse temperature Stickel plots with linear fits in the region $T \leq 1$. *Left Panel:* $3dKA$ model. *Right Panel:* $3dR10$ model. The x-intercepts give the values of $1/T_{VFT}$.

culated using the data for the relaxation times as follows.

$$\Phi(T) \equiv \left(\frac{d\ln\tau_\alpha}{d(1/T)}\right)^{-\frac{1}{2}}. \tag{5}$$

It is easy to see that

$$\Phi(T) = \sqrt{K_{VFT}T_{VFT}}\left(\frac{1}{T_{VFT}} - \frac{1}{T}\right), \tag{6}$$

if the VFT form holds. This implies that a plot of $\Phi(T)$ versus $1/T$ should be linear and extrapolate to zero at $1/T_{VFT}$. Thus T_{VFT} can be obtained from a linear fit to the $\Phi(T)$ versus $1/T$ data and the fragility parameter K_{VFT} can be obtained from the slope of the fitted straight line.

Stickel plots (plots of $\Phi(T)$ versus $1/T$) for the two model systems considered here are shown in Fig. 8. It is clear from these plots, even without doing any fitting, that the data for larger values of ρ_{pin} would extrapolate to zero at larger values of $1/T$, yielding lower values of T_{VFT}. This is precisely the trend observed in the values of T_{VFT} obtained from the VFT plots shown in the main paper. Also, the Stickel plots in Fig. 8 do not show any indication of this trend changing at still lower temperatures.

We fitted the low temperature parts ($T \leq 1$) of the $\Phi(T)$ data to straight lines to get T_{VFT} and K_{VFT}. These fits are shown in Fig. 8. The values obtained (shown in Fig. 9) agree with those obtained from our

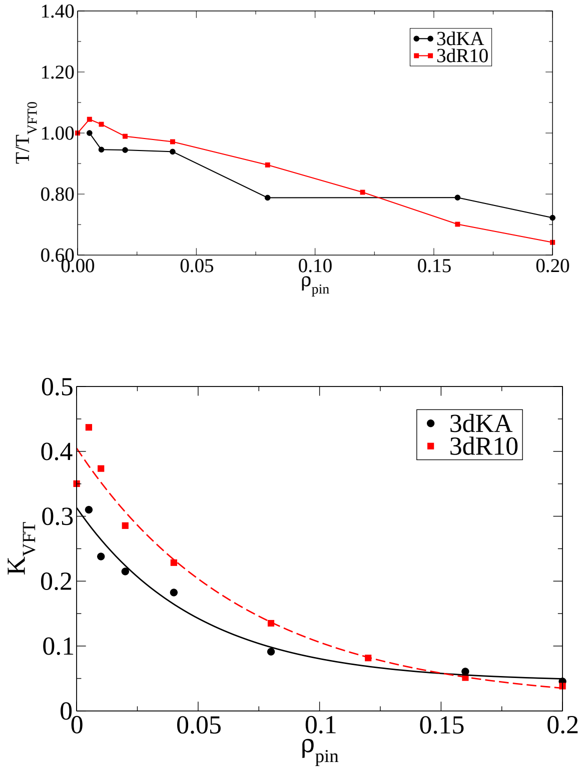

FIG. 9: Fit parameters obtained from fits in Fig. 8. *Left Panel:* T_{VFT}. *Right Panel:* K_{VFT}.

direct fits within error bars and show exactly the same general dependence on the pinning density.

[1] W. Kob and H. C. Andersen, Phys. Rev. E **51**, 4626 (1995).
[2] C. Cammarota and G. Biroli, Proc. Nat. Acad. Sci. **109**, 8850 (2012).
[3] F. Stickel, E. W. Fischer and R. Richert, J. Chem. Phys., **102**, 6251, (1995).
